# Cutting through the value chain: the long-run effects of decoupling the East from the West

**DOI:** 10.1007/s10663-022-09561-w

**Published:** 2023-01-18

**Authors:** Gabriel Felbermayr, Hendrik Mahlkow, Alexander Sandkamp

**Affiliations:** 1 Austrian Institute of Economic Research (WIFO), Vienna University of Economics and Business & CESifo, Vienna, Austria; 2 Kiel Institute for the World Economy (IfW), Kiel, Germany; 3 University of Kiel (CAU), Kiel Institute (IfW), CESifo & KCG, Wilhelm-Seelig-Platz 1, 24118 Kiel, Germany

**Keywords:** Trade, Non-tariff barriers, Global value chains, Quantitative trade model, China, Russia, European Union, F11, F13, F14, F17

## Abstract

With ever-increasing political tensions between China and Russia on one side and the EU and the US on the other, it only seems a matter of time until protectionist policies cause a decoupling of global value chains. This paper uses a computable general equilibrium trade model calibrated with the latest version of the GTAP database to simulate the effect of such a decoupling–implemented by doubling non-tariff barriers–between the two blocks on trade and welfare. Imposing import barriers almost completely eliminates bilateral imports. In addition, changes in price levels lead to higher imports and lower exports of the imposing country group from and to the rest of the world. The targeted country group increases exports to the rest of the world and reduces imports. Welfare falls in all countries involved, suggesting that governments should strive to cooperate rather than turn away from each other. By imposing a trade war on Russia, the political West could inflict severe damage on the Russian economy because of the latter’s smaller relative economic size.

## Introduction

Global value chains are currently being attacked on several fronts. The two most prominent threats are political. First, the COVID-19 pandemic has revealed the vulnerability of international value chains, prompting politicians to push for a re-shoring of production in order to reduce dependence on foreign suppliers and thus improve crisis resilience of the domestic economy.^1^ The second threat stems from increased political tensions between China and Russia on one side, and the EU and the US on the other. The Russian invasion of Ukraine on 24th February 2022 has pushed the relationship between Russia and the political West to a new low, having provoked a cascade of economic sanctions and counter-sanctions.

Political struggles with China seem negligible in comparison. However, while the Sino-American trade war has raged for several years (Bown 2020), relations between China and the EU are also by no means trouble-free. Following the opening of a Taiwanese representative office in Lithuania’s capital Vilnius in November 2021, both Lithuanian firms and European companies using Lithuanian inputs complained about trade restrictions with China (European Commission 2022). As a response, the EU has launched a case against China at the WTO, while simultaneously continuing the development of an Anti-Coercion Instrument. This recent spat has only been the latest in a series of conflicts between China and the EU. They are spurring a tendency to decouple, as both the EU and China are turning inwards to reduce their dependence on each other.^2^

Against this background, we use a computable general equilibrium model of international trade based on Caliendo and Parro (2015) to investigate the impact of five decoupling scenarios on trade and welfare. Incorporating intra- and international input–output linkages, the model quantifies the effects of changes in bilateral trade barriers on 65 sectors in 141 countries, covering 98 % of economic activity worldwide. It is calibrated using the most recent version 10 of the input–output-database of the Global Trade Analysis Project (GTAP) as described by Aguiar et al. (2019) and allows quantifying both direct and indirect trade effects such as trade diversion and real income effects.

In the model, decoupling is achieved by a doubling of non-tariff barriers (NTBs) which strongly reduces trade while not completely eliminating it.^3^ The first scenario assumes a doubling in NTBs between the EU and China (both unilateral and reciprocal). Keeping in mind the ongoing conflicts between China and the US, the second scenario analyses a decoupling between China on one side and the US and its allies (including the EU) on the other.^4^ In light of the crisis in Ukraine, Scenario 3 simulates the effects of a trade war between Russia and the US and its allies. Scenario 4 models an even broader divide between the EU on one side and Brazil, Russia, India and China (BRIC) on the other. Scenario 5 investigates a trade dispute between the US allies and the BRIC countries.

The paper shows that a unilateral decoupling of China from the EU (i.e. a doubling of NTBs on Chinese imports from the EU) would almost eliminate bilateral imports - a phenomenon the literature calls trade destruction (Bown and Crowley 2007). Perhaps less straightforward, Chinese exports to both the EU and the rest of the world also fall. This is because NTBs increase the cost of imported intermediates in China, thus reducing competitiveness of Chinese exporters that rely on foreign inputs. In addition, falling Chinese demand for EU products leads to a real depreciation of the Euro (in the model through falling EU prices), further reducing the competitiveness of Chinese exports relative to European goods.

Our model also makes predictions on how trade relations of both parties with third countries would evolve. In line with the literature (Bown and Crowley 2007), we find that NTBs imposed on Chinese imports from the EU cause trade deflection (EU exports to other countries increase), import source diversion (China imports more from non-EU countries) and trade depression (the EU imports less from the rest of the world). A unilateral decoupling by the EU inverts these results. Not surprisingly, both parties would lose from a trade war (reciprocal imposition of NTBs), with welfare declining by 0.92 and 0.78 % in China and the EU respectively. Engaging in a trade war with the US and its allies (including the EU) would be even more costly for China.

A trade war between Russia and the political West would inflict high economic damage on Russia, while the US and its allies would remain relatively unharmed on average. However, welfare declines are unevenly distributed, with Eastern European countries suffering most (even though still less than Russia). Overall, it becomes clear that relative economic size matters both for maximizing the welfare loss suffered by the political rival and for minimizing the own party’s losses.

The paper relates to three strands of literature. First, it contributes to the literature investigating the impact of NTBs on trade. Several studies provide evidence for the trade dampening effects of NTBs, implying that they would constitute an effective instrument to achieve decoupling. By estimating ad-valorem tariff equivalents, Kee et al. (2009) show that NTBs restrict trade by almost as much as tariffs. Hoekman and Nicita (2011) even find a stronger trade dampening effect of NTBs compared to tariffs. In particular, the authors show that a 10 % increase in NTBs is associated with a 1.7 % reduction in trade. Similar conclusions are reached by Bouët et al. (2008) as well as Bratt (2017). Following the increased use of NTBs, the overall level of protection has not decreased between 1997 and 2015 despite the fall in tariffs during that period (Niu et al. 2017).

Ghodsi et al. (2017) investigate different types of NTBs, estimating trade dampening effects ranging between 5 and 30 %, depending on the type of NTB imposed. More recently, Kinzius et al. (2019) show that NTBs reduce imports of affected products by up to 12 %, with certain types of NTBs having an even stronger effect on trade.^5^ We add to this literature by modelling how the reduction in trade induced by the extreme measure of doubling NTBs translates into welfare changes. Crucially, we employ “exact hat algebra” (Dekle et al. 2008) to solve the model in changes rather than levels. This avoids the difficult endeavour of quantifying initial NTBs (Egger et al. 2015), which depend on manifold policy instruments.

Sanctions are a specific form of NTBs. Through an embargo, products become non-tradable across country pairs. The counterfactual analysis of the impact of sanctions on trade flows is often modelled by introducing non-tradable sectors (Etkes and Zimring 2015; Crozet and Hinz 2020; Hinz and Monastyrenko 2022). We show that a doubling of NTBs acts almost as an embargo, even though it does not completely reduce trade flows to zero across country pairs. In an extension, we increase NTBs on energy imports from Russia up until they are completely eliminated. We also demonstrate that the damage inflicted by trade restrictions on the strategic rival increases with the economic size of the countries implementing them.^6^

The paper also relates to Amiti et al. (2019) and Fajgelbaum et al. (2020) who investigate the impacts of the recent waves of protectionism. While those studies focus on the impact of tariffs recently imposed by the US government, this paper investigates how NTBs could be used to seal off an economy from a particular trading partner.

Second, the paper contributes to the literature investigating the effect of trade barriers on untargeted countries. These are trade deflection as countries targeted by trade barriers export more to third countries (Bown and Crowley 2006, 2007, 2010; Baylis and Perloff 2010), import source diversion as countries imposing barriers on imports increase imports from non-targeted countries (Konings et al. 2001; Baylis and Perloff 2010) and trade depression as targeted countries reduce imports from non-targeted countries (Bown and Crowley 2007). Incorporating all these phenomena through changes in relative prices, our model reveals the impacts of a trade war between China and the EU not only on the two economies but also on their trading partners.

Third, the paper relates to the literature on modelling trade flows in computable general equilibrium models (Costinot and Rodríguez-Clare 2014). These models have their theoretical foundation in the so-called gravity equation of international trade (Yotov et al. 2016). They model the structure of bilateral trade flows as a function of bilateral costs. In doing so, these models can be solved in changes, a practice made famous by Dekle et al. (2008) and referred to as “exact hat algebra”. The model used builds on the Ricardian framework developed by Eaton and Kortum (2002) and extended by Caliendo and Parro (2015) to incorporate input–output linkages between multiple sectors. This framework is especially useful for our analysis because input–output linkages play an important role in enhancing the effects of trade policy.^7^

We include services trade and NTBs in this framework in a fashion similar to Felbermayr et al. (2021, 2020). Unlike them, we use the latest version of GTAP (Aguiar et al. 2019) for the calibration of the model. GTAP has the advantage that it not only contains a higher sectoral resolution (65 sectors) but also more countries (121 countries and 20 aggregate regions) than e.g. the World Input–Output Database (WIOD). Therefore, the model is based on detailed input–output linkages among a wide range of sectors and countries. Given the important role played by intermediate products in our model, the paper also relates to Gopinath and Neiman (2014); Halpern et al. (2015); Eaton et al. (2016); Alfaro et al. (2019); Antràs and Gortari (2020) as well as more generally to Goldberg et al. (2010) and Antràs (2020).

This paper is not the first to simulate the impact of an increase in NTBs on trade and welfare. Sforza and Steininger (2020) model the welfare effects of the COVID-19 induced shock to global production networks in both an open economy with current trade cost levels and a closed economy which is characterized by 100 percentage points higher trade costs (the same increase as the one we use). Eppinger et al. (2021) apply a similar approach, showing that the welfare loss resulting from a COVID shock is smaller in a de-globalised world. Both papers show that welfare is lower in a world with high trade barriers.

Instead of modelling a global decoupling, this paper investigates the impact of NTBs that are imposed between two very specific groups of countries. In contrast to Bachmann et al. (2022) and Chepeliev et al. (2022), who simulate the effects of decoupling from Russian energy exports, we investigate the impacts of a general decoupling that is not limited to energy trade. By revealing the true cost of an escalating trade war between China and Russia on one side and the EU and the US on the other, the findings are highly relevant for policymakers.

The remainder of the paper is structured as follows: Sect. 2 describes the model used for the analysis, while Sect. 3 provides an overview of the data used to calibrate the model. Section 4 presents the baseline results, followed by extensions and a discussion in Sect. 5. Sect. 6 concludes.

## Model

The analysis is carried out with the help of the “Kiel Institute Trade Policy Evaluation” model (“KITE model”) which is based on the trade model proposed by Caliendo and Parro (2015), who provide a multi-sector version of the Eaton and Kortum (2002) gravity model with input–output linkages.

### Setup

There are *N* countries, indexed *o* and *d*, and *J* sectors, indexed *j* and *k*. Production uses one aggregate factor, which we consider as *labour*.^8^ The factor is mobile across sectors $L_d=\sum _{j}^{J}L_{d}^{j}$, but not across countries. All markets are perfectly competitive. Sectors are either wholly tradable or non-tradable. In each sector, there is a continuum of goods $\omega ^{j}\in [0,1]$. Households in *d* obtain utility from consumption *C* according to the following two-tier Cobb-Douglas utility function:

$$\begin{aligned} u_d=\prod _{j\in {\mathcal {J}}}\left( \exp \int _0^1\ln C_d(\omega ^{j})d\omega ^{j}\right) ^{\alpha _{d}^{j}}, \end{aligned}$$

where $\alpha$ is the constant sectoral expenditure share and $\sum _{j\in {\mathcal {J}}}\alpha _{d}^{j}=1$. Household income $I_{d}$ is derived from the supply of labour $L_{d}$ at wage $w_{d}$ and a lump-sum transfer of tariff revenues. Goods are produced using labour *l* and *composite* intermediate input bundles *m* from all sectors. Countries differ in their productivity for different goods from the continua, inversely captured by the input requirement *z*, and the input cost shares $\gamma$. The production technology is Cobb-Douglas:

$$\begin{aligned} q_{o}^{j} (\omega ^{j}) = \left[ z_{o}^{j} (\omega ^{j})\right] ^{-1} \left[ l_{o}^{j} (\omega ^{j}) \right] ^{\beta _{o}^{j}} \left[ \prod _{k = 1}^{J} m_{o}^{k,j} (\omega ^{j}) ^{\gamma _{o}^{k,j}} \right] ^{1 - \beta _{o}^{j}} \end{aligned}$$

where $\beta _{o}^{j} \in [0, 1]$ is the cost share of labour and $\gamma _{o}^{k,j} \in [0, 1]$ with $\sum _{k} \gamma _{o}^{k,j} = 1$ the share of sector *k* in sector *j*’s intermediate. Overall efficiency of a producer is denoted by $z_{o}^{j} (\omega ^{j})$ and labour input by $l_{o}^{j} (\omega ^{j})$. Intermediate input bundles $m_{o}^{k,j} (\omega ^{j})$ from sector *k* used to produce $\omega ^{j}$ are themselves Cobb-Douglas composites:

$$\begin{aligned} m_d^{j}=\exp \int _0^1 \ln d_d(\omega ^j)d\omega ^j, \end{aligned}$$

where $d_d(\omega ^j)$ is the demand for the specific variety $\omega ^j$ as intermediate inputs. Unit costs (which equal the price due to perfect competition and constant returns to scale) are given by $c_{o}^{j}z_{o}(\omega ^{j})$, where the cost of the input bundles are given by

1 $$\begin{aligned} c_{o}^{j} = \Upsilon _{o}^{j} w_{o}^{\beta _{o}^{j}} \left[ \prod _{k = 1}^{J} (P_{o}^{k})^{\gamma _{o}^{k,j}} \right] ^{1 - \beta _{o}^{j}} \end{aligned}$$

where $P_{o}^{k}$ is the price of a composite intermediate good from sector *k*, and the constant $\Upsilon _{o}^{j} = \prod _{k = 1}^{J} (\gamma _{o}^{k,j} - \beta _{o}^{j} \gamma _{o}^{k,j})^{-\gamma _{o}^{k,j} + \beta _{o}^{j} \gamma _{o}^{k,j}} (\beta _{o}^{j} \gamma _{o}^{j})^{-\beta _{o}^{j} \cdot \gamma _{o}^{j}}$. Hence, the cost of the input bundle depends on wages and the prices of *all* composite intermediate goods in the economy. A firm in country *o* can supply its output to country *d* at price

2 $$\begin{aligned} p_{od}^{j} = \phi _{od}^{j} \cdot \frac{c_{o}^{j}}{z_{o}^{j}(\omega ^{j})} \end{aligned}$$

where $\phi _{od}^{j}$ denote generic bilateral sector-specific trade frictions.^9^ These can take a variety of forms — e.g. tariffs, non-tariff barriers, export taxes. In that case we can specify

$$\begin{aligned} \phi _{od}^{j}&= \tau _{od}^{j} \cdot \kappa _{od}^{j} \cdot \zeta _{od}^{j} \cdot NTB_{od}^{j}, \end{aligned}$$

where $\tau _{od}^{j}$ represent sector-specific ad-valorem tariffs, $\kappa _{od}^{j} \ge 1$ iceberg trade costs, $\zeta _{od}^{j}$ export taxes or subsidies, and $NTB_{od}^{j} \ge 1$ non-tariff barriers.^10^

Producers of sectoral composites in country *d* search for the supplier with the lowest cost across all possible origin locations, i.e.

3 $$\begin{aligned} p_{d}^{j}&= \min _{o} \left\{ p_{od}^{j} \right\} . \end{aligned}$$

Ricardian comparative advantage is induced à la Eaton and Kortum (2002) through a country-specific idiosyncratic productivity draw $z^{j}$ from a Fréchet distribution.^11^ As Caliendo and Parro (2015) show, the price of the composite good is then given as

4 $$\begin{aligned} P_{d}^{j}&= A^{j} \left[ \sum _{o = 1}^{N} \lambda _{o}^{j} ( c_{o}^{j} \phi _{od}^{j})^{-\theta ^{j}} \right] ^{-1 / \theta ^{j}} \end{aligned}$$

where $A^{j} = \Gamma (\xi ^{j})^{1 / (1 - \sigma ^{j})}$ is a constant with $\Gamma (\xi ^{j})$ being a Gamma function evaluated at $\xi ^{j} = 1 + (1 - \sigma ^{j})/\theta ^{j}$ and $\sigma ^{j}$ is the elasticity of substitution between different goods in the continua of sector *j*.

Total expenditures on goods from sector *j* in country *d* are given by $X_{d}^{j} = P_{d}^{j} Q_{d}^{j}$. The expenditure on those goods originating from country *o* is called $X_{od}^{j}$, such that the share of *j* from *o* in *d* is $\pi _{od}^{j} = X_{od}^{j} / X_{d}^{j}$. In other words, it is the share of an exporter country in the total expenditure, by sector, of an importer country. This share can also be expressed as

5 $$\begin{aligned} \pi _{od}^{j} = \frac{\lambda _{o}^{j} ( c_{o}^{j} \phi _{od}^{j})^{-\theta ^{j}}}{\sum _{h = 1}^{N} \lambda _{h}^{j} ( c_{h}^{j} \phi _{hd}^{j})^{-\theta ^{j}}} \end{aligned}$$

which forms the core of a gravity equation.

### General equilibrium

Total expenditures $X_{d}^{j}$ on goods from sector *j* are the sum of the firms’ and households’ expenditures on the composite intermediate good, either as input to production or for final consumption

6 $$\begin{aligned} X_{d}^{j} = \sum _{k = 1}^{J} (1 - \beta _{d}^{k}) \gamma _{d}^{j,k} \sum _{o = 1}^{N} X_{o}^{k} \frac{\pi _{do}^{k}}{\tau _{do}^{k} \zeta _{do}^{k}} + \alpha _{d}^{j} I_{d} \end{aligned}$$

with $I_{d} = w_{d} L_{d} + R_{d} + D_{d}$, i.e., labour income, government revenue (tariff and export taxes minus export subsidies) and the aggregate trade balance. The first term on the right-hand side gives demand of sectors *k* in all countries *o* for intermediate usage of sector *j* varieties produced in country *d*, the second term denotes final demand. Sectoral trade balance is simply the difference between imports and exports

7 $$\begin{aligned} D_{d}^{j} = \sum _{o = 1}^{N} X_{od}^{j} - X_{do}^{j} \end{aligned}$$

and the aggregate trade balance $D_{d} = \sum _{j = 1}^{J} D_{d}^{j}$, and $\sum _{d = 1}^{N} D_{d} = 0$, with $D_{d}$ being exogenously and $D_{d}^{j}$ being endogenously determined.^12^ The trade balance can then be expressed as

8 $$\begin{aligned} \sum _{j = 1}^{J} \sum _{o = 1}^{N} X_{d}^{j} \frac{\pi _{od}^{j}}{\tau _{od}^{j} \zeta _{od}^{j}} - D_{d} = \sum _{j = 1}^{J} \sum _{o = 1}^{N} X_{o}^{j} \frac{\pi _{do}^{j}}{\tau _{do}^{j} \zeta _{do}^{j}}. \end{aligned}$$

The goods market clearing (6) and trade balance (8) conditions close the model.

### Comparative statics in general equilibrium

We are interested in the effects of different decoupling scenarios on trade flows and welfare (measured as real income). Decoupling is introduced via doubling of non-tariff barriers of all imports from a specific trading partner. In order to quantify the comparative static effects of changes in non-tariff barriers on trade flows and welfare, we solve the model in changes, as suggested by Dekle et al. (2008). Let *x* denote the initial level of a variable and $x^{\prime }$ its counterfactual level. Then, trade cost shocks are given by ${\hat{x}}_{od}^{j} = x_{od}^{j \prime } / x_{od}^{j}$. In our analysis where we only consider a change in non-tariff barriers—leaving all other trade costs unchanged, as e.g. tariffs—this leads to ${\hat{\phi }}_{od}^{j} = NTB_{od}^{j \prime } / NTB_{od}^{j} = 2$ for country *d* that decouples from imports of country *o* in sector *j*.^13^ In a similar fashion as iceberg trade costs, non-tariff barriers are $\ge 1$. For example, if importer *d* decouples from exporter *o* and the initial $NTB_{od}^{j} = 1.1$, a 100% increase in non-tariff barriers yields a counterfactual $NTB_{od}^{j \prime } = 2.2$. The change in welfare is

9 $$\begin{aligned} {\hat{W}}_d = \frac{{\hat{I}}_{d}}{\prod _{j=1}^{J}({\hat{P}}_{d}^{j})^{\alpha _{d}^{j}}}. \end{aligned}$$

In Appendix C, we present the system of equations in changes required to solve the model.

### Overview of the scenarios

We consider five scenarios: (1) A decoupling of the EU 27 and China; (2) A decoupling of the US and its allies from China; (3) A decoupling of the US and its allies from Russia; (4) A decoupling of the EU 27 from the BRIC countries; and (5) A decoupling of the US and its allies from the BRIC countries. In principle, decoupling–i.e. shifting production away from the trading partner and back to the own economy–can be achieved without government intervention if firms decide to shift their production back home. It can also be enforced by the government through import bans, prohibitively high tariffs or NTBs such as state aid or public procurement policies. Within the model, decoupling cannot be implemented explicitly. Instead, it is modelled by a doubling in NTBs on imports (i.e. an increase by 100 percentage points) in all sectors relative to current levels.^14^ As shown in Sect. 4, this results in a strong reduction in imports without completely eliminating them.^15^

Exports remain untreated, as it is assumed that countries want to reduce their import dependence only. This assumption is not realistic in case of the sanctions imposed against Russia, as the EU and the US also impose restrictions on their exports to Russia. Such behaviour is captured by the scenario simulating an increase in Russian NTBs. Within the model, imposing NTBs on, say, the EU’s exports to Russia has the same effect as Russia imposing NTBs on its imports from the EU.

Each scenario is divided into three sub-scenarios that simulate a unilateral decoupling (modelled by an increase in NTBs on imports of the decoupling country) as well as a trade war (both countries increasing NTBs on each other’s imports). For example, the three sub-scenarios of Scenario 1 simulate a unilateral decoupling by the EU vis-à-vis China (i.e. a doubling in NTBs on EU imports from China, Scenario 1A), a unilateral decoupling by China vis-à-vis the EU (i.e. a doubling in NTBs on Chinese imports from the EU, Scenario 1B) as well as a reciprocal decoupling / trade war (i.e. a doubling in NTBs on EU imports from China and a doubling in NTBs on Chinese imports from the EU, Scenario 1C).

For each sub-scenario, we simulate changes in bilateral trade flows between the trading partners (measuring trade destruction), changes in exports to the rest of the world (trade deflection), changes in imports from the rest of the world (import source diversion and trade depression), the change in total exports as well as the change in welfare. For country groups such as the EU or BRIC, the welfare change is computed as an average across countries, weighted by the share of a single country’s value-added within the group (before the imposition of NTBs). The change in trade flows is calculated by the groups’ sum of respective real trade flows before and after decoupling. Real flows are calculated by dividing nominal trade flows by the sectoral price change in the destination.

## Data

To simulate the effects of a (simultaneous) decoupling of trading partners in general equilibrium, we need to identify the model parameters. Consumption shares and input coefficients ($\alpha$, $\beta$, and $\gamma$), as well as bilateral trade shares ($\pi$), value added (*wL*), and initial trade imbalances (*D*) are obtained from the GTAP input–output table (Aguiar et al. 2019). The latest version of GTAP provides data for the year 2014.^16^ We choose GTAP because of its rich geographical (121 countries and 20 aggregated regions) and sectoral (65 sectors) coverage. In contrast to the World Input Output Database (WIOD, used for example by Bachmann et al. (2022)), GTAP includes separate fossil fuels sectors such as gas, coal and oil. This makes the model particularly well suited to analyse decoupling from Russia. For a full list of all countries, see Appendix D.

Model outcomes crucially depend on the productivity dispersion parameters $\theta ^{j}$. Therefore, we take well established gravity estimates from the literature (Fontagné et al. 2018).^17^ For the service sectors, we rely on an estimate for the aggregate service sector provided by Egger et al. (2012).

**Fig. 1:**
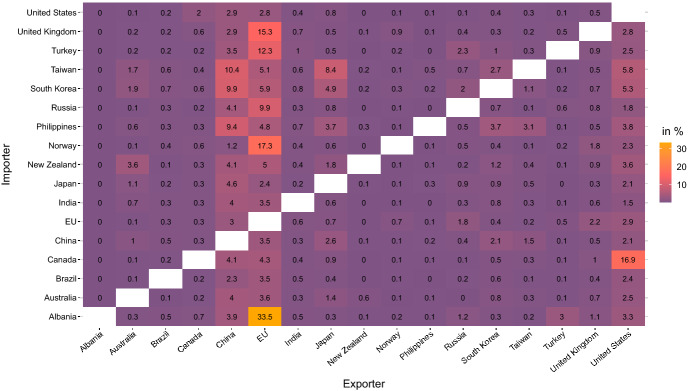
Bilateral imports in % of importer GDP. Note: Own calculation based on 2014 values of the GTAP 10 database

In Sect. 4 we show that relative economic size matters for the welfare loss following decoupling. The extent of relative economic size becomes clear when looking at bilateral imports in % of the importer’s GDP in Fig. 1. Russia’s imports from the EU equal almost 10 % of its GDP, while for the EU, imports from Russia only amount to 1.8 % of its GDP. When the political West forms a coalition, the imbalance becomes even more severe. Russia’s imports from the US and its allies equal 15.2 % of its GDP, while for the US and its allies, imports from Russia only amount to 2.7 % of their joint GDP.^18^

## Results

### Scenario 1: Decoupling between China and the EU

Table 1 Panel A presents the results for a decoupling between the EU and China (Scenario 1). A unilateral decoupling of the EU from China almost completely eliminates bilateral imports (Scenario 1A). More specifically, EU imports from China fall by 95.82 % (Column 1). In contrast, Chinese exports to the rest of the world increase by 8.22 % (Column 3) as Chinese exporters find alternative markets following the increase in the cost of exporting to the EU (trade deflection). Nevertheless, the increased exports to the rest of the world are unable to fully compensate for the loss in export business to the EU, so that overall, Chinese exports fall by 8.49 % (Column 7). This is accompanied by a reduction in welfare of 0.55 % in China (Column 9).

**Table 1:** Scenarios 1, 2, and 3, changes in percent

**Panel A: Scenario 1** “EU-China decoupling”										
Decoupling scenario	$\Delta$ Bilateral exports		$\Delta$ Exports to RoW		$\Delta$ Imports from RoW		$\Delta$ Total exports		$\Delta$ Welfare	
	China (1)	EU (2)	China (3)	EU (4)	China (5)	EU (6)	China (7)	EU (8)	China (9)	EU (10)
1A EU	− 95.82	− 15.92	8.22	− 5.30	− 8.62	6.43	− 8.49	− 6.43	− 0.55	− 0.58
1B China	− 10.31	− 97.35	− 5.57	5.42	7.16	− 4.03	− 6.33	− 5.49	− 0.46	− 0.28
1C Bilateral	− 96.21	− 97.70	2.25	− 0.49	− 2.22	2.27	− 13.56	− 10.81	− 0.92	− 0.78

**Panel B: Scenario 2** “US allies-China decoupling”										
	China (1)	US al. (2)	China (3)	US al. (4)	China (5)	US al. (6)	China (7)	US al. (8)	China (9)	US al. (10)
2A US al.	− 93.90	− 44.84	48.75	− 9.68	− 37.67	11.66	− 35.43	− 19.92	− 2.44	− 0.79
2B China	− 34.46	− 95.54	− 26.45	11.88	45.09	− 8.27	− 31.18	− 19.42	− 2.10	− 0.49
2C Bilateral	− 95.74	− 97.32	11.70	− 0.75	− 10.76	3.88	− 51.70	− 28.88	− 3.55	− 0.95

**Panel C: Scenario 3** “US allies-Russia decoupling										
	Russia (1)	US al. (2)	Russia (3)	US al. (4)	Russia (5)	US al. (6)	Russia (7)	US al. (8)	Russia (9)	US al. (10)
3A US al.	− 95.68	− 39.68	107.35	− 1.59	− 34.19	3.19	− 28.74	− 3.80	− 7.30	− 0.13
3B Russia	− 27.53	− 96.22	− 22.51	2.03	50.58	− 1.11	− 25.87	− 3.67	− 4.71	− 0.09
3C Bilateral	− 96.36	− 97.69	58.79	− 0.06	− 9.09	1.90	− 45.21	− 5.72	− 9.71	− 0.17

Perhaps less straightforward, EU exports to China also decline, albeit to a lesser extent (15.92 %, Column 2 of Table 1 Panel A). This result is driven by two mechanisms. First, NTBs imposed by the EU against China reduce EU demand for Chinese products. In addition to falling bilateral imports, this leads to falling prices of Chinese products.^19^ This real depreciation makes Chinese products more attractive relative to European ones, thus reducing EU exports to China. In addition, falling prices in China also increase competitiveness of Chinese products in the rest of the world, resulting in the aforementioned trade deflection (Column 3) as well as trade depression, i.e. a fall in Chinese imports from the rest of the world (Column 5).^20^

Second, increasing prices of imports from China due to NTBs mean that EU imports are diverted away from China and towards other countries. In fact, EU imports from the rest of the world increase by 6.43 % (import source diversion, Column 6). At the same time, some production shifts from China to Europe. Production is thus shifted to less productive producers outside China. This decrease in specialisation increases average production costs of affected goods. In particular, more expensive intermediate products also increase production costs of companies in the EU and consequently reduce their international competitiveness, causing a fall in exports (and rise in imports). Consequently, EU exports to the rest of the world fall by 5.3 % (Column 4) so that overall, European exports decline by 6.43 % (Column 8). Welfare in the EU falls by 0.58 % (Column 10).

A unilateral decoupling by China has exactly opposite effects (Scenario 1B). Chinese imports from the EU fall by 97.43 % (Column 2), while Chinese imports from the rest of the world increase by 7.16 % (Column 5). Following the fall in competitiveness caused by higher prices of intermediates and a real depreciation of the Euro relative to the Renminbi, Chinese exports to the EU (the rest of the world) fall by 10.31 % (5.57 %, Columns 1 and 3). Overall, Chinese exports fall by 6.33 % (Column 7) in this scenario, resulting in a welfare loss of 0.46 % (Column 9).

Meanwhile, European exports are deflected to the rest of the world (an increase of 5.42 %, Column 4). Overall, EU exports nevertheless decline by 5.49 % (Column 8). At the same time, falling EU prices reduce imports from the rest of the world (Column 6). The EU experiences a welfare loss of 0.28 % (Column 10). The EU thus suffers less from a restriction on its exports to China (Scenario 1B) than on its imports (Scenario 1A). This is not surprising given the trade deficit the EU has with China.

A full-blown trade war between China and the EU (Scenario 1C) puts trade between the two economies almost to a complete standstill. Chinese exports (imports) to (from) the EU decline by 96.2 % (97.7 %) in this scenario (Columns 1 and 2). For China with its trade surplus with the EU, downward pressure on prices resulting from a fall in demand from the EU outweighs the loss in competitiveness due to higher prices of intermediates, so that Chinese exports to the rest of the world increase by 2.25 % (Column 3), while imports fall by 2.22 % (Column 5).

For the EU with its trade deficit vis-à-vis China, higher prices of intermediates from China dominate the negative price effects resulting from falling demand from China. Consequently, EU exports to the rest of the world decline by 0.49 % (Column 4), while imports increase by 2.27 % (Column 6). Overall, Chinese and EU exports fall by 13.56 % and 10.81 % respectively (Columns 7 and 8). Welfare declines by 0.92 % in China (Column 9) and 0.78 % in the EU (Column 10).

**Fig. 2:**
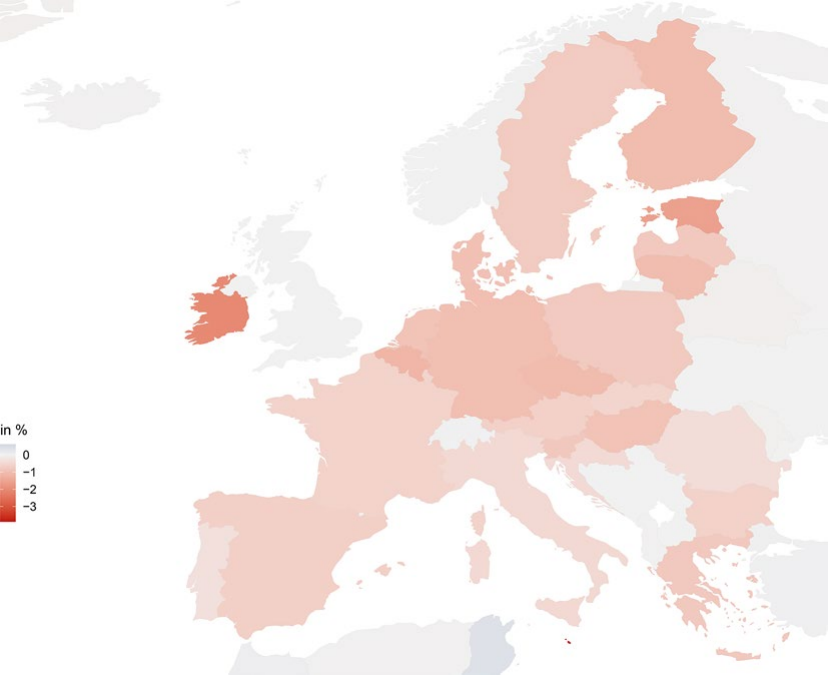
Welfare effects by European country in Scenario 1C, changes in %

A trade war thus harms both China and Europe. However, welfare losses are not evenly distributed, as Fig. 2 shows. Within the EU, small open economies such as Malta (−3.89 %), Ireland (−2.04 %) and Estonia (−1.59 %) lose most.^21^ Outside the EU, most countries remain relatively unaffected, as pictured in Fig. 6 in the Appendix. For example, welfare in Russia falls by 0.02 %. A few countries such as Cambodia (+0.62 %), Bangladesh (+0.44 %) and Tunisia (+0.38 %) even slightly gain from a trade war between China and the EU. Welfare gains for the US a negligible (+0.005 %).

### Scenario 2: Decoupling between China and US allies

Scenario 2 models a decoupling of China from the US and its allies, which for the purposes of this paper are defined as the EU27, Albania, Australia, Canada, Iceland, Japan, New Zealand, Norway, Philippines, South Korea, Taiwan, Turkey, and the United Kingdom.^22^ The results are reported in Table 1 Panel B, which is structured in the same way as Table 1 Panel A. The effects of decoupling on bilateral trade between China and the US and its allies are qualitatively similar to Scenario 1. Bilateral imports of the party imposing NTBs are almost eliminated completely, while bilateral exports also fall (Columns 1 and 2 of Table 1 Panel B). The decline in bilateral exports of the imposing party is stronger than in Scenario 1 because the US and its allies constitute a larger market for Chinese products than just the EU. Consequently, the fall in demand for Chinese products is larger, causing larger real adjustments in the bilateral exchange rate.

Another reason for the strong decline in bilateral exports can potentially be found in the production networks China has with several countries among the US allies, in particular its neighbours (Aichele and Heiland 2018). Global supply chains are characterised by multiple border crossings (Johnson and Noguera 2017). If a value chain for a particular product crosses borders between China and the US allies several times, NTBs imposed on US allies’ imports can have strong impacts on their exports, too. For example, presume that a product is initially produced in China, then exported to South Korea where it is combined with another input before being sent back to China for final manufacturing. If South Korea imposes NTBs on imports from China, this also reduces its exports. With multiple border crossings not uncommon in modern value chains, NTBs imposed on imports can have strong impacts on a country’s exports to the partner country that is targeted by the trade restrictions. This is particularly true for the country group US allies, as it includes several countries with close trade relationships with China.

In Scenario 2A (B), driven by price adjustments, we observe trade deflection of Chinese (US allies’) exports following the imposition of NTBs by US allies (China, Columns 3 and 4). Trade depression is also present, as imports by the targeted country from the rest of the world decline (Columns 5 and 6). On the other hand, following the fall in competitiveness of products produced with the help of intermediates subject to NTBs as well as real price adjustment following shifts in relative demand, exports by the implementing country group to both the targeted country and the rest of the world decline (Columns 1 to 4), while imports from the rest of the world increase (import source diversion, Columns 5 and 6). Effects on overall exports and welfare are negative for both parties (Columns 7 to 10). As shown in Scenario 2C, China loses three times more from a trade war (−3.55 %, Column 9) than the US and its allies (−0.95 %, Column 10). This is not surprising as the share of Chinese exports going to this country group is larger than China’s share in US allies’ exports, in particular as US allies trade a lot among themselves.

### Scenario 3: Decoupling between Russia and US allies

Against the background of the crisis in Ukraine, a decoupling by the US and its allies from Russia seems particularly relevant. Scenario 3 thus simulates the effects of an increase in NTBs between the US and its allies on one side and Russia on the other. Qualitatively, the results, presented in Table 1 Panel C, are similar to those in Scenario 2. However, the magnitude of the welfare effects differs strongly from those experienced in a trade war between the US allies and China. The US and its allies are a much bigger trade partner for Russia than Russia is for the US and its allies. Even when just considering the EU, Russia only accounted for 4.8 % of the EU’s total trade in 2020, while the EU accounted for 37.3 % of Russian trade (European Commission 2022). Consequently, a unilateral imposition of NTBs by the US and its allies against imports from Russia (Scenario 3A) hits Russia much harder (7.3 % drop in welfare, Column 9) than the imposing countries (0.13 % fall, Column 10).

Export barriers imposed by the US and its allies against Russia are captured by Scenario 3B.^23^ Restricting exports to Russia does less economic harm than restricting imports from the country. However, welfare in Russia still falls by 4.71 % in this scenario (Column 9). A trade war, i.e. a restriction on both Russia’s exports and imports (Scenario 3C), reduces welfare in Russia by 9.71 % (Column 9) but only by 0.17 % in the US and its allies (Column 10). Overall, a trade war with Russia is much less costly for the US and its allies than a trade war with China. Once again, this is not surprising given that China accounted for 22 % of the EU’s imports and 10 % of its exports in 2021, whereas Russia accounted for less than 7 % of EU imports and 4 % of exports (UN 2022).

**Table 2:** Scenarios 4, 5, and 6, changes in percent

**Panel A: Scenario 4** “EU-BRIC decoupling”										
Decoupling scenario	$\Delta$ Bilateral exports		$\Delta$ Exports to RoW		$\Delta$ Imports from RoW		$\Delta$ Total exports		$\Delta$ Welfare	
	BRIC (1)	EU (2)	BRIC (3)	EU (4)	BRIC (5)	EU (6)	BRIC (7)	EU (8)	BRIC (9)	EU (10)
4A EU	− 95.96	− 21.75	13.19	− 8.88	− 10.20	15.44	− 11.48	− 11.56	− 0.84	− 1.05
4B BRIC	− 16.09	− 96.96	− 7.08	11.14	9.98	− 8.11	− 9.12	− 11.31	− 0.83	− 0.58
4C Bilateral	− 96.48	− 97.57	4.79	0.35	− 1.62	6.24	− 18.10	− 19.99	− 1.45	− 1.42

**Panel B: Scenario 5** “US allies-BRIC decoupling”										
	BRIC (1)	US al. (2)	BRIC (3)	US al. (4)	BRIC (5)	US al. (6)	BRIC (7)	US al. (8)	BRIC (9)	US al. (10)
5A US al.	−93.75	−47.85	55.02	−14.10	−38.12	22.12	−39.25	−28.36	−2.75	−1.10
5B BRIC	−37.77	−95.41	−25.16	19.65	43.66	−13.14	−33.15	−28.95	−2.43	−0.70
5C Bilateral	−95.72	−97.42	16.10	−0.46	−10.82	8.35	−54.75	−41.41	−3.86	−1.32

**Panel C: Scenario 6** “US allies-Russia bilateral energy decoupling”										
	Russia (1)	US al. (2)	Russia (3)	US al. (4)	Russia (5)	US al. (6)	Russia (7)	US al. (8)	Russia (9)	US al. (10)
6 Energy	−56.69	−26.82	56.08	−0.46	−22.57	2.01	−19.51	−1.99	−6.62	−0.10

**Fig. 3:**
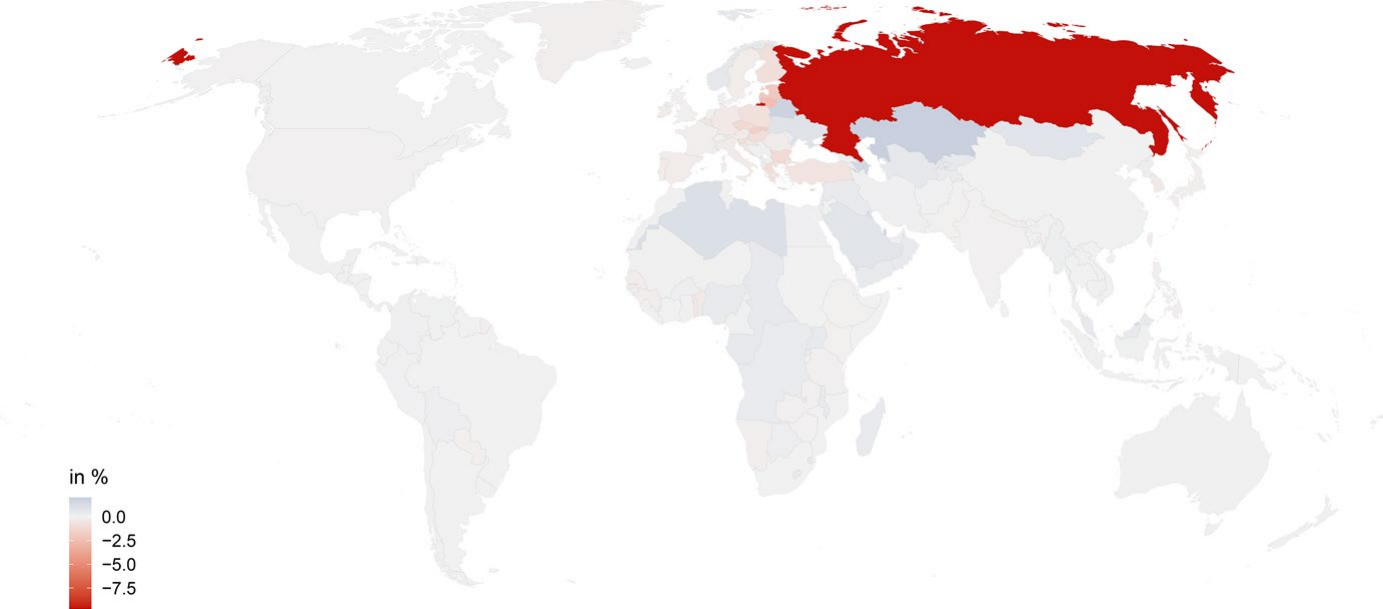
Welfare effects in Scenario 3C, changes in %

Within the EU, the welfare effects following a trade war with Russia are, however, quite unevenly distributed, as Fig. 3 shows.^24^ Eastern European countries lose most from such a conflict. In the Baltic States, welfare declines by 2.48 % in Lithuania, 2.02 % in Latvia and 1.98 % in Estonia. Slovakia (−1.68 %), the Czech Republic (−1.16 %) and Bulgaria (−1.11 %) also experience above average declines in welfare. Even countries with limited direct exposure suffer because of indirect effects. For example, Russia is neither amongst the top ten export destinations nor amongst the top ten origin countries of Slovenia (Statistical Office Slovenia 2022). Nevertheless, the country suffers above average welfare effects (−0.8 %) in Scenario 3C because of indirect links with Russia via its European trading partners. Outside Europe, welfare in the US declines by 0.04 %. China even profits slightly from a conflict between Russia and the US allies (+0.02 %).

### Scenario 4: Decoupling between BRIC and the EU

Scenario 4 models a trade conflict between the EU on one side and Brazil, Russia, India and China on the other. The aim of this Scenario is to investigate the consequence of China and Russia “teaming up” with other large emerging economies. The purchases of Russian oil by China and India in the first half of 2022 (Bruegel 2022) may constitute a first step in this direction. The results, presented in Table 2 Panel A, are qualitatively similar to Scenarios 1 and 2. Bilateral imports of the trading partner imposing the NTB are almost eliminated (Columns 1 and 2). Exports of the country group subject to NTBs are deflected to the rest of the world, while exports of the implementing country group decline (Columns 3 and 4). In contrast, imports from the rest of the world into the imposing country group increase (import source diversion), while those into the targeted country group decline (trade depression, Columns 5 and 6). Total exports as well as welfare fall in both country groups (Columns 7 to 10). In terms of foregone welfare, the EU suffers more from a trade war with the BRIC countries than from a decoupling purely from China.

### Scenario 5: Decoupling between BRIC and US allies

Scenario 5 splits the world into the US and its allies on one side and the BRIC countries on the other. As in the other scenarios, bilateral trade falls drastically following the imposition of NTBs (Columns 1 and 2 of Table 2 Panel B). Trade deflection, import source diversion and trade depression are also present (Columns 3 to 6). Total exports of affected parties drop in all three sub-scenarios (Columns 7 and 8) and are accompanied by a fall in welfare in both the BRIC countries ($-$3.86 %, Column 9) and US allies ($-$1.32 %, Column 10).

**Fig. 4:**
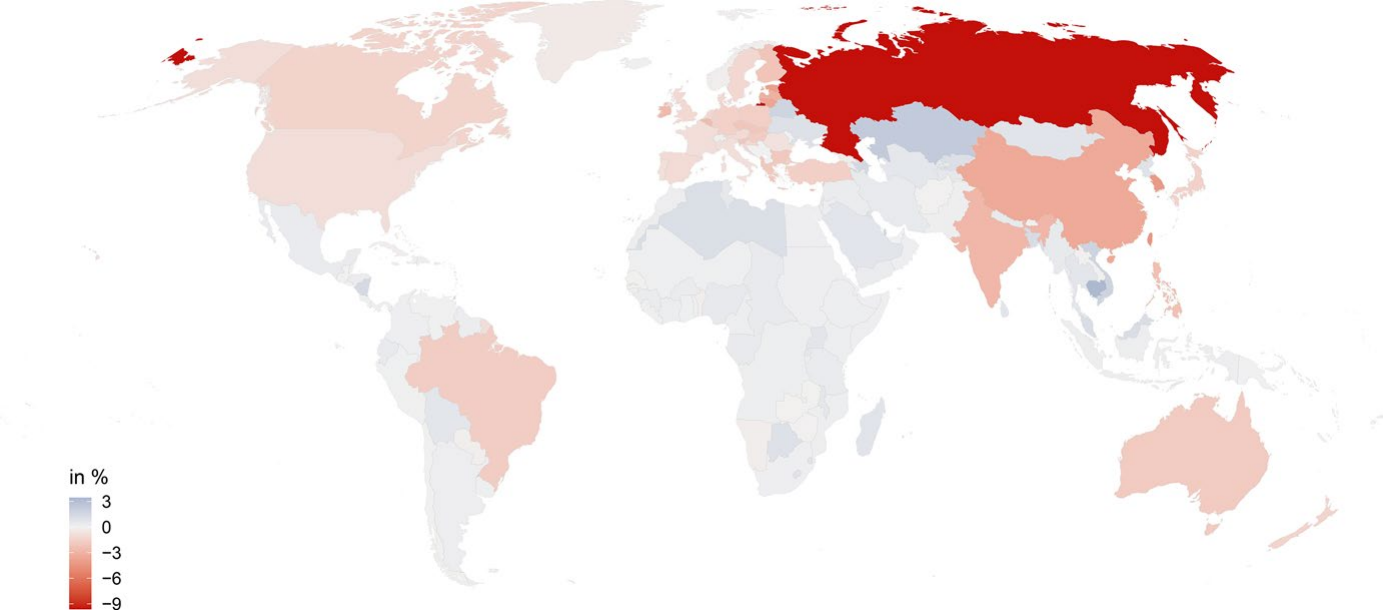
Welfare effects in Scenario 5C, changes in %

Figure 4 takes a closer look at Scenario 5C and illustrates the welfare changes for each country following a trade war between the US allies and the BRIC countries. Welfare losses are not evenly distributed. Within the BRIC countries, Russia loses by most (−9.62 %), followed by China (−3.5 %), India (−2.84 %) and Brazil (−1.75 %). Among the US allies, small open economies that are strongly interlinked with China experience the highest welfare losses. These are first and foremost Taiwan (−4.43 %), South Korea (−4.25 %) and Japan (−1.53 %). Within the EU, Malta (−6.34 %), Estonia (−3.95 %) and Lithuania (−3.67 %) are most strongly affected.^25^ The US experience a 0.91 % welfare loss.

## Extensions

### Decoupling between Russia and US allies - energy sector

In an extension, we simulate the effect of an embargo by the EU, the US and its allies on Russian energy exports only. In the model, NTBs on these countries’ imports of coal, gas, oil and petroleum products from Russia are increased until trade in these sectors is completely eliminated.^26^ Results are presented in Table 2 Panel C below. Since energy products make up a large proportion of Russian exports, it is not surprising to see bilateral exports from Russia to the EU, the US and its allies fall by almost 57 % (Column 1). Russian exports to the rest of the world would increase by 56 % in the model (Column 3). This is in line with developments in the first half of 2022, which witnessed increasing Russian exports of oil to China and India (Bruegel 2022). However, the drop in exports to the political West cannot be compensated completely, meaning that total Russian exports fall by 19.5 % (Column 7) and welfare declines by 6.6 % (Column 9). Compared with the welfare decline of 7.3 % for Russia in Scenario 3A (doubling of NTBs by the EU, the US and its allies on all Russian exports) this reiterates the importance of energy exports for the Russian economy.

The EU, the US and its allies are only mildly affected, experiencing welfare declines of 0.1 % on average. Welfare in Germany - Europe’s largest economy - is expected to decline by 0.3 %. This effect is in a similar order of magnitude, albeit slightly smaller than the lower bound of 0.5 % estimated by Bachmann et al. (2022). As discussed in Subsection 5.3, this difference results from the different time-horizon of our model.

### Varying levels of NTBs

Within the model, decoupling is simulated by doubling NTBs because this strongly reduces bilateral trade without completely eliminating it. In doing so, we follow other papers such as Sforza and Steininger (2020), who characterize a closed economy as one in which trade costs are doubled. Of course 100 percentage points may seem somewhat arbitrary, and we could as well have chosen 90 or 110 percentage points. As an extension to Scenario 1C, we therefore model a continuous increase in NTBs between the EU and China. Different degrees of mutual decoupling are simulated, with NTB increases ranging from 5 to 100 percentage points.

**Fig. 5:**
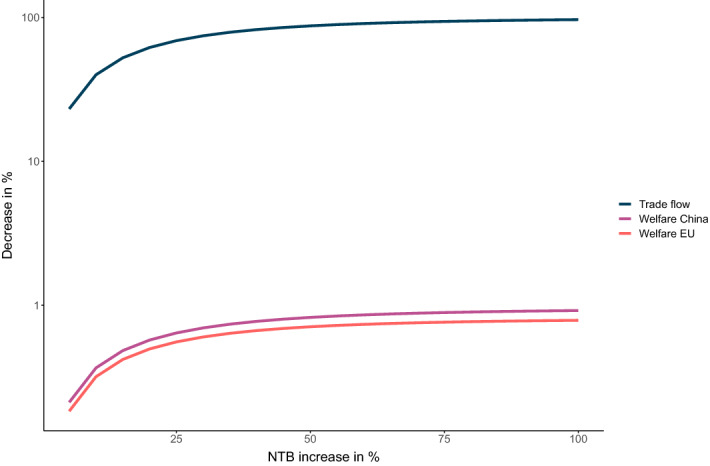
EU–China bilateral decoupling for NTB increases of 5 to 100 percentage points. Note: Decreases of bilateral trade flows (imports and exports) and welfare in %. Y-axis is log transformed

**Fig. 6:**
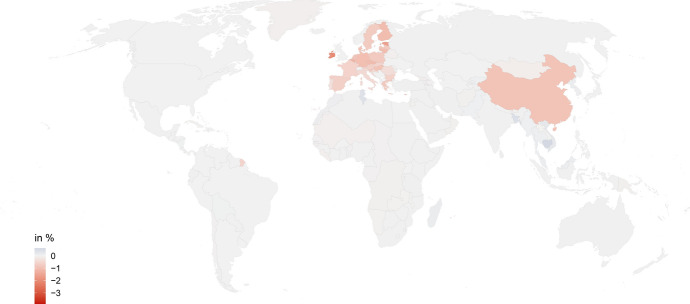
Welfare effects by country in Scenario 1C, changes in %

Changes in bilateral trade flows and welfare are depicted in Fig. 5. The functions are strictly concave and approach their maxima quickly. An NTB increase of 50 percentage points already decreases bilateral trade flows by 87.64 %, compared to 96.87 % following an NTB increase of 100 percentage points. Welfare falls by 0.71 and 0.82 % for the EU and China in case of an NTB increase of 50 percentage points, compared to 0.78 and 0.92 % respectively for a 100 percentage points increase in NTBs. Our baseline results are thus not sensitive to whether NTBs are increased by 90 or 100 percentage points.

### Limitations of the model

When considering the policy implications of our model results, it is important to stress that we estimate long-run effects in a comparative statics framework. We thus compare the status quo equilibrium with a counterfactual equilibrium in a decoupled world. With respect to energy trade, this means that new infrastructure such as pipelines or LNG terminals have already been constructed. If decoupling were to happen within a short period of time, many firms would find themselves being cut-off from their suppliers, prices of affected products may skyrocket, and several firms may be forced to temporarily suspend production until supply chains adjust. The current decoupling between the political West and Russia is a case in point. While our estimates suggest only mild long run effects for the West (−0.17 % on average, −0.4 % for Germany) short run effects are expected to be stronger (0.5 to 3 % reduction in German GDP, (Bachmann et al. 2022)). In fact, GDP forecasts already had to be adjusted more severely (for example a reduction of 3.5 percentage points in the estimated growth rate for Germany in 2023, (Holtemöller et al. 2022)). The short-term effects on welfare can thus be expected to be much more negative than our estimated long-run effects.

A second limitation of the model lies in the fact that it assumes a certain degree of substitutability across sourcing countries. In principle, all goods can be produced in all countries, albeit at varying production costs. While this assumption is realistic at the sectoral level, it is certainly not true at the product level. The assumption is less restrictive in the long-run, although certain raw materials cannot be substituted within a policy relevant time horizon.

In line with many Ricardian trade models, we only use one aggregate factor of production. The factor is a composite of many factors, as for example labour, capital, and land, which are all non-tradeable in our model. In a similar framework, Costinot and Rodríguez-Clare (2014) have shown that the introduction of the additional factor capital, which is not only fully mobile across sectors but also across countries, slightly increases gains from trade.

In the context of a decoupling between the East and the West, an unrestricted movement of capital between the country blocks seems unrealistic. In order to avoid the difficult endeavour of setting the right amount of rigidity in cross-country capital movements, we follow the most restrictive variant and include capital in our aggregate non-tradeable production factor. However, even though capital products are not modelled explicitly, intermediate goods can in fact be interpreted as capital goods. For example, cars used as inputs in the agricultural sector can be seen as capital goods that are also tradable internationally.

## Conclusion

Since the early 2000s, the political landscape has shifted away from ever closer market integration through global trade and towards a decoupling - if not break up - of global value chains. Perhaps most worrying, tensions between China on one side and the EU and the US on the other could tear apart value chains that have added so much to economic growth. In order to contribute to a better understanding of the impacts these actions could have on both trade and welfare, this paper has modelled the effect of such a divide of the world with NTBs on trade and welfare. Employing a general equilibrium trade model calibrated with the latest version of GTAP, we simulate the effects of five decoupling scenarios on 121 countries and 20 regions, taking into account detailed input–output linkages among 65 economic sectors.

Within the model, a Chinese increase in NTBs against EU exports by 100 percentage points exhibits all the trade effects that are already well documented in the empirical literature: First trade destruction, i.e. an almost complete elimination of Chinese imports from the EU. Second import source diversion, i.e. an increase in Chinese imports from the rest of the world following a fall in competitiveness of Chinese firms due to higher prices of intermediates and a real exchange rate appreciation. For the same reason, China also exports less to both the EU and the rest of the world. Third trade deflection, as EU exports are deflected to other countries following a fall in EU prices and fourth, trade depression, i.e. a fall in EU imports from the rest of the world, also following lower EU prices. Welfare declines in both economies following such a unilateral decoupling by China. The above results are reversed if the EU decoupled from China instead.

A trade war, in which both China and the EU raise their bilateral NTBs, reduces welfare in China and the EU by 0.92 and 0.78 %, respectively. The rest of the world mainly remains unaffected by such a conflict. The model also shows that forging alliances increases the damages inflicted on the strategic rival. In particular, a trade war between China and the US and its allies (including the EU) incurs a welfare loss of 3.55 % for China, while the welfare decline for the US and its allies only amounts to 0.95 %. The same is true - albeit to a lesser extent - for a trade war between the BRIC countries and the EU. Welfare losses to the EU amount to 1.42 % in this scenario, while the BRIC countries suffer a loss of 1.45 % on average.

Our findings also offer a lesson for the conflict between Russia and the West. As relative size matters, trade restrictions are more harmful for the target if more countries implement them. For the same reason, Russia would lose much more from a trade war with the West than China. Specifically, a reciprocal decoupling of Russia from the US and its allies reduces welfare in Russia by 9.71 %. In contrast, welfare of US allies remains almost unaffected (−0.17 %), although Eastern European countries lose substantially (up to 2.48 % in the case of Lithuania). In fact, implementing countries often experience smaller welfare losses if they act together. Increasing economic sanctions thus comes at relatively low costs for the US and its allies on average, at least in the long run. Small Eastern European countries which lose more could be compensated. Finally, a trade war between the US and its allies on one side and the BRIC countries on the other would reduce welfare in both country groups by 1.32 % and 3.86 % respectively.

Teaming up can thus increase the harm imposed on the strategic rival. However, if one country group decouples, it is never the best option for the other country group to retaliate, as this would increase the cost for both parties. Overall, the simulation results confirm what economic intuition would dictate: Intentionally dividing the world with non-tariff barriers would reduce welfare in all countries involved in the conflict and should thus never be done light-heartedly.

## Appendix

### A: Further results

See Tables 3, 4, 5.

**Table 3:** Scenario 1C, changes in %

Country	$\Delta$ Welfare	$\Delta$ Total		$\Delta$ Exports		$\Delta$ Imports	
		Exports	Imports	China	RoW	China	RoW
	(1)	(2)	(3)	(4)	(5)	(6)	(7)
Austria	−0.63	−2.92	−2.98	−97.79	−0.16	−96.59	1.95
Belgium	−1.17	−3.56	−3.34	−98.17	−0.22	−96.28	1.39
Bulgaria	−0.62	−2.40	−2.13	−97.05	−0.84	−96.75	1.00
China	−0.92	−13.56	−17.14		2.25		−2.22
Cyprus	−0.86	−3.36	−.17	−98.40	2.11	−97.01	−0.11
Czechia	−1.06	−2.87	−3.49	−97.08	−4.17	−95.81	5.03
Germany	−1.00	−6.40	−7.07	−97.71	0.20	−96.20	2.44
Denmark	−0.95	−4.40	−4.82	−97.35	0.96	−95.60	1.16
Spain	−0.66	−3.53	−4.27	−97.69	−0.99	−95.87	2.54
Estonia	−1.59	−3.23	−3.84	−97.46	−2.20	−96.49	2.49
Finland	−1.04	−4.93	−5.41	−97.89	3.64	−96.22	0.44
France	−0.60	−4.54	−4.57	−97.24	−0.84	−96.13	2.31
Greece	−0.79	−3.07	−3.07	−98.19	−1.92	−95.68	2.26
Croatia	−0.45	−1.48	−1.58	−98.09	−0.49	−96.48	1.02
Hungary	−0.92	−3.90	−4.40	−97.93	−2.74	−96.47	3.19
Ireland	−2.04	−3.79	−5.63	−98.30	2.28	−97.47	0.39
Italy	−0.47	−4.05	−4.30	−97.08	−2.10	−96.27	2.70
Lithuania	−1.03	−1.63	−2.03	−96.52	−1.85	−96.39	2.01
Luxembourg	−0.56	−3.35	−2.79	−98.15	0.55	−97.33	−2.03
Latvia	−0.78	−1.21	−1.55	−95.60	−1.54	−96.24	3.29
Malta	−3.89	−7.59	−8.87	−98.37	−4.89	−97.71	16.56
Netherlands	−0.90	−2.92	−3.89	−97.71	−1.85	−96.14	3.98
Poland	−0.77	−2.44	−2.88	−98.01	−2.84	−96.15	4.03
Portugal	−0.34	−2.17	−1.71	−98.72	−1.22	−96.56	1.41
Romania	−0.39	−1.28	−1.29	−97.66	−2.62	−96.43	3.34
Slovakia	−0.55	−3.55	−2.50	−99.35	−2.99	−96.09	4.27
Slovenia	−0.81	−1.46	−1.93	−97.61	−1.29	−96.59	3.60
Sweden	−0.76	−3.54	−3.96	−97.73	1.71	−96.14	0.76

**Table 4:** Scenario 3C, changes in %

Country	$\Delta$ Welfare	$\Delta$ Total		$\Delta$ Exports		$\Delta$ Imports	
		Exports	Imports	Russia	RoW	Russia	RoW
	(1)	(2)	(3)	(4)	(5)	(6)	(7)
Albania	−0.36	−0.84	−1.05	−98.36	−2.20	−86.47	2.65
Australia	0.07	0.18	0.48	−98.69	−2.72	−97.29	0.56
Austria	−0.28	−1.90	−1.77	−97.45	1.35	−97.09	0.21
Belgium	−0.51	−2.37	−2.28	−97.58	−1.07	−95.36	2.41
Bulgaria	−1.11	−5.44	−5.73	−97.86	−3.30	−96.76	11.08
Canada	0.01	−0.18	−0.09	−97.84	−0.56	−96.72	−0.05
China	0.02	0.13	0.36	−2.52	0.67	96.43	−3.32
Cyprus	−1.15	−7.39	−6.74	−98.77	3.46	−96.20	0.02
Czechia	−1.16	−3.41	−4.15	−97.48	1.40	−99.39	7.82
Germany	−0.40	−2.42	−2.86	−97.48	0.97	−96.83	2.52
Denmark	−0.08	−1.55	−1.34	−97.59	−0.99	−92.68	0.54
Spain	−0.22	−1.48	−1.58	−97.71	0.85	−93.89	2.52
Estonia	−1.98	−12.84	−11.90	−95.42	−5.50	−92.55	−1.27
Finland	−0.88	−6.82	−7.93	−97.37	2.55	−97.01	2.93
France	−0.16	−1.40	−1.26	−97.94	1.19	−93.05	0.22
United Kingdom	−0.09	−1.48	−1.15	−98.43	−0.71	−95.12	0.66
Greece	−0.95	−9.36	−8.45	−98.07	−8.65	−92.14	10.30
Croatia	$-$0.49	$-$4.49	$-$4.67	$-$98.15	$-$2.08	$-$95.69	9.21
Hungary	$-$0.86	$-$5.95	$-$6.99	$-$97.27	$-$2.41	$-$98.65	9.56
Ireland	$-$0.32	$-$0.67	$-$0.83	$-$98.68	1.51	$-$96.86	$-$0.58
Italy	$-$0.31	$-$2.40	$-$2.95	$-$96.49	0.62	$-$98.33	11.13
Japan	$-$0.14	$-$1.38	$-$1.63	$-$98.44	0.31	$-$99.06	3.48
South Korea	$-$0.36	$-$1.99	$-$2.41	$-$97.95	$-$0.12	$-$96.13	2.57
Lithuania	$-$2.48	$-$14.12	$-$13.86	$-$95.77	$-$6.13	$-$98.70	35.82
Luxembourg	$-$0.33	$-$1.84	$-$1.61	$-$98.58	0.96	$-$97.47	$-$1.38
Latvia	$-$2.02	$-$7.28	$-$7.35	$-$97.24	$-$1.88	$-$94.32	15.98
Malta	$-$1.05	$-$3.08	$-$2.90	$-$98.93	0.37	$-$86.67	$-$0.70
Netherlands	$-$0.12	$-$2.74	$-$3.22	$-$97.10	$-$2.97	$-$96.75	4.85
Norway	0.55	0.86	3.22	$-$98.64	$-$9.77	$-$94.28	6.75
New Zealand	$-$0.04	$-$0.60	$-$0.58	$-$97.57	0.27	$-$98.85	0.48
Philippines	$-$0.13	$-$0.76	$-$0.81	$-$98.16	0.16	$-$99.08	0.90
Poland	$-$0.78	$-$6.08	$-$6.38	$-$97.39	$-$1.94	$-$98.36	10.85
Portugal	$-$0.25	$-$0.96	$-$1.06	$-$98.27	0.99	$-$90.18	1.98
Romania	$-$0.28	$-$3.37	$-$3.27	$-$96.93	$-$1.11	$-$96.85	5.31
Russia	$-$9.71	$-$45.21	$-$64.00		58.79		$-$9.09
Slovakia	$-$1.68	$-$6.01	$-$6.74	$-$98.57	1.64	$-$97.90	11.58
Slovenia	$-$0.83	$-$3.05	$-$3.25	$-$97.95	2.83	$-$95.58	$-$0.66
Sweden	$-$0.26	$-$1.98	$-$2.22	$-$97.66	1.14	$-$98.03	4.10
Turkey	$-$0.63	$-$4.99	$-$4.84	$-$97.17	0.00	$-$91.19	2.64
Taiwan	$-$0.22	$-$0.93	$-$1.30	$-$97.17	$-$0.42	$-$94.38	0.07
United States	$-$0.04	$-$1.33	$-$1.00	$-$98.36	0.16	$-$92.96	$-$0.45

**Table 5:** Scenario 5C, changes in %

Country	$\Delta$ Welfare	$\Delta$ Total		$\Delta$ Exports		$\Delta$ Imports	
		Exports	Imports	BRIC	RoW	BRIC	RoW
	(1)	(2)	(3)	(4)	(5)	(6)	(7)
Albania	$-$1.94	$-$7.33	$-$6.44	$-$99.15	$-$1.81	$-$93.77	6.03
Australia	$-$1.87	$-$18.99	$-$22.73	$-$98.68	18.04	$-$95.45	$-$1.22
Austria	$-$1.06	$-$5.53	$-$5.18	$-$97.66	0.25	$-$96.59	5.24
Belgium	$-$2.59	$-$9.31	$-$8.40	$-$98.57	$-$1.98	$-$95.77	7.43
Bulgaria	$-$1.94	$-$7.80	$-$7.54	$-$97.67	$-$6.39	$-$96.52	16.76
Brazil	$-$1.75	$-$43.60	$-$39.66		$-$5.12		13.05
Canada	$-$1.43	$-$6.06	$-$7.38	$-$96.14	$-$3.08	$-$95.19	13.96
China	$-$3.50	$-$51.32	$-$64.34		12.91		$-$13.83
Cyprus	$-$2.37	$-$13.08	$-$11.94	$-$98.60	6.52	$-$96.35	0.38
Czechia	$-$2.10	$-$6.32	$-$7.20	$-$97.56	$-$4.73	$-$97.01	27.74
Germany	$-$1.55	$-$10.36	$-$11.09	$-$97.72	$-$0.41	$-$96.08	10.92
Denmark	$-$1.27	$-$7.12	$-$7.17	$-$97.75	$-$1.74	$-$94.73	5.37
Spain	$-$1.07	$-$6.95	$-$7.64	$-$97.80	$-$1.48	$-$94.73	7.87
Estonia	$-$3.95	$-$16.14	$-$15.77	$-$96.44	$-$9.02	$-$94.07	6.40
Finland	$-$2.16	$-$13.98	$-$15.32	$-$97.73	5.17	$-$96.55	9.98
France	$-$0.94	$-$7.30	$-$6.91	$-$97.69	$-$1.30	$-$95.29	6.16
United Kingdom	$-$1.18	$-$10.60	$-$9.51	$-$98.58	$-$4.74	$-$95.64	8.65
Greece	$-$1.98	$-$14.42	$-$12.86	$-$98.26	$-$9.68	$-$93.26	16.19
Croatia	$-$1.18	$-$6.54	$-$6.88	$-$98.25	$-$3.80	$-$95.67	17.04
Hungary	$-$1.76	$-$9.91	$-$11.03	$-$97.86	$-$6.51	$-$97.61	28.38
India	$-$2.84	$-$37.77	$-$32.50		17.51		$-$9.87
Ireland	$-$2.68	$-$5.83	$-$7.72	$-$98.51	2.67	$-$97.30	2.84
Italy	$-$0.89	$-$7.77	$-$8.02	$-$97.01	$-$4.02	$-$96.75	24.38
Japan	$-$1.53	$-$24.72	$-$23.21	$-$97.25	3.84	$-$95.91	5.03
South Korea	$-$4.25	$-$27.85	$-$28.50	$-$97.29	8.07	$-$95.18	$-$3.40
Lithuania	$-$3.67	$-$15.97	$-$16.00	$-$96.24	$-$11.27	$-$98.07	63.82
Luxembourg	$-$2.24	$-$6.30	$-$5.43	$-$98.38	0.61	$-$97.35	$-$2.59
Latvia	$-$3.16	$-$8.79	$-$9.31	$-$97.29	$-$5.89	$-$94.69	29.96
Malta	$-$6.34	$-$12.08	$-$13.26	$-$98.63	$-$4.66	$-$96.10	18.56
Netherlands	$-$1.28	$-$6.78	$-$8.09	$-$97.81	$-$6.74	$-$95.71	16.66
Norway	$-$0.13	$-$3.49	$-$2.47	$-$98.08	$-$10.08	$-$95.46	10.29
New Zealand	$-$1.40	$-$14.13	$-$15.32	$-$95.06	9.48	$-$95.69	0.71
Philippines	$-$2.37	$-$22.24	$-$17.73	$-$97.46	$-$2.13	$-$94.92	9.04
Poland	$-$1.66	$-$8.66	$-$9.28	$-$97.74	$-$7.27	$-$97.03	29.37
Portugal	$-$0.83	$-$4.70	$-$4.01	$-$97.86	$-$2.84	$-$94.45	6.58
Romania	$-$0.75	$-$5.17	$-$4.70	$-$97.59	$-$6.55	$-$96.07	15.13
Russia	$-$9.62	$-$45.12	$-$63.59		50.88		$-$16.95
Slovakia	$-$2.08	$-$9.08	$-$8.34	$-$99.01	$-$4.18	$-$97.12	30.29
Slovenia	$-$2.10	$-$5.09	$-$5.97	$-$97.91	$-$0.86	$-$94.28	7.33
Sweden	$-$1.21	$-$7.19	$-$7.53	$-$97.77	1.39	$-$96.65	11.29
Turkey	$-$1.62	$-$11.41	$-$10.72	$-$97.81	$-$6.55	$-$93.70	12.43
Taiwan	$-$4.43	$-$25.44	$-$30.13	$-$97.17	18.63	$-$96.34	$-$10.37
United States	$-$0.91	$-$17.05	$-$14.61	$-$96.47	$-$3.73	$-$95.62	9.92

### B: Additional figures

See Fig. 6.

### C: Solving for counterfactual equilibria

As suggested by Dekle et al. (2008), a counterfactual general equilibrium for alternative trade costs in the form of ${\hat{\phi }}_{od}^{j} = \phi _{od}^{j \prime } / \phi _{od}^{j}$ — i.e. where any variable ${\hat{x}}$ denotes the relative change from a previous value *x* to a new one $x'$ — can be solved for in changes such that

A1 $$\begin{aligned} \text {\textit{Input costs}} \qquad {\hat{c}}_{d}^{j}&= {\hat{w}}_{d}^{\beta _{d}^{j}} \left( \prod _{k = 1}^{J} [ {\hat{P}}_{d}^{k} ]^{\gamma _{d}^{k,j}} \right) ^{1 - \beta _{d}^{j}} \end{aligned}$$

A2 $$\begin{aligned} \text {\textit{Prices}} \qquad {\hat{P}}_{d}^{j}&= \left( \sum _{o = 1}^{N} \pi _{od}^{j} [ {\hat{\phi }}_{od}^{j} {\hat{c}}_{o}^{j} ]^{-1 / \theta ^{j}} \right) ^{-\theta ^{j}}\end{aligned}$$

A3 $$\begin{aligned} \text {\textit{Trade shares}} \qquad \pi _{od}^{j \prime }&= \pi _{od}^{j} \left( \frac{{\hat{c}}_{o}^{j}}{{\hat{P}}_{d}^{j}} {\hat{\phi }}_{od}^{j} \right) ^{-1 / \theta ^{j}}\end{aligned}$$

A4 $$\begin{aligned} \text {\textit{Expenditures}} \quad X_{d}^{j \prime }&= \sum _{k = 1}^{J} (1 - \beta _{d}^{k}) \gamma _{d}^{j,k} \left( \sum _{o = 1}^{N} \frac{\pi _{do}^{k \prime }}{\tau _{do}^{k \prime } \zeta _{do}^{k \prime }} X_{o}^{k \prime } \right) + \alpha _{d}^{j} I_{d}^{\prime } \quad \text {with} \end{aligned}$$

A5 $$\begin{aligned} \text {\textit{Income, value added}} \qquad I_{d}^{\prime }&= {\hat{w}}_{d} w_{d} L_{d} \nonumber \\ \text {\textit{Tariff revenue}} \quad \qquad&\qquad + \sum _{k = 1}^{J} \sum _{o = 1}^{N} (\tau _{od}^{k \prime } - 1) \left( \frac{\pi _{od}^{k \prime }}{\tau _{od}^{k \prime }}\right) X_{d}^{k \prime } \nonumber \\ \text {\textit{Export tax revenue}} \quad \qquad&\qquad + \sum _{k = 1}^{J} \sum _{o = 1}^{N} (\zeta _{do}^{k \prime } - 1) \left( \frac{\pi _{do}^{k \prime }}{\tau _{do}^{k \prime } \zeta _{do}^{k \prime }}\right) X_{o}^{k \prime } \nonumber \\ \text {\textit{Trade balance}} \quad \qquad&\qquad - D_{d}^{\prime } \nonumber \\ \text {\textit{Trade balance}} \qquad D_{d}&= \sum _{j = 1}^{J} \sum _{o = 1}^{N} \frac{\pi _{od}^{j \prime }}{\tau _{od}^{j \prime } \zeta _{od}^{j \prime }} X_{d}^{j \prime } - \sum _{j = 1}^{J} \sum _{o = 1}^{N} \frac{\pi _{do}^{j \prime }}{\tau _{do}^{j \prime } \zeta _{do}^{j \prime }} X_{o}^{j \prime }. \end{aligned}$$

### D: Data

#### D.1: List of countries included in the model

Albania; United Arab Emirates; Argentina; Armenia; Australia; Austria; Azerbaijan; Belgium; Benin; Burkina Faso; Bangladesh; Bulgaria; Bahrain; Belarus; Bolivia; Brazil; Brunei; Botswana; Canada; Switzerland; Chile; China; Cote d’Ivoire; Cameroon; Colombia; Costa Rica; Cyprus; Czechia; Germany; Denmark; Dominican Republic; Ecuador; Egypt; Spain; Estonia; Ethiopia; Finland; France; United Kingdom; Georgia; Ghana; Guinea; Greece; Guatemala; Hong Kong SAR China; Honduras; Croatia; Hungary; Indonesia; India; Ireland; Iran; Israel; Italy; Jamaica; Jordan; Japan; Kazakhstan; Kenya; Kyrgyzstan; Cambodia; South Korea; Kuwait; Laos; Sri Lanka; Lithuania; Luxembourg; Latvia; Morocco; Madagascar; Mexico; Malta; Mongolia; Mozambique; Mauritius; Malawi; Malaysia; Namibia; Nigeria; Nicaragua; Netherlands; Norway; Nepal; New Zealand; Oman; Pakistan; Panama; Peru; Philippines; Poland; Puerto Rico; Portugal; Paraguay; Qatar; Romania; Russia; Rwanda; Saudi Arabia; Senegal; Singapore; El Salvador; Slovakia; Slovenia; Sweden; Togo; Thailand; Tajikistan; Trinidad & Tobago; Tunisia; Turkey; Taiwan; Tanzania; Uganda; Ukraine; Uruguay; United States; Venezuela; Vietnam; South Africa; Zambia; Zimbabwe.

#### D.2: List of regions included in the model

1. South Central Africa: Angola, Democratic Republic of the Congo.
2. Rest of Central America: Belize.
3. Rest of Caribbean: Anguilla, Antigua and Barbuda, Aruba, Bahamas, Barbados, British Virgin Islands, Cayman Islands, Cuba, Dominica, Grenada, Haiti, Montserrat, Netherlands Antilles, Saint Kitts and Nevis, Saint Lucia, Saint Vincent and Grenadines, Turks and Caicos Islands, Virgin Islands (US).
4. Rest of Central Africa: Central African Republic, Chad, Congo, Equatorial Guinea, Gabon, Sao Tome and Principe.
5. Rest of East Asia: Korea, Democratic People’s Republic of, Macao, Special Administrative Region of China.
6. Rest of Eastern Africa: Burundi, Comoros, Djibouti, Eritrea, Mayotte, Seychelles, Somalia, Sudan.
7. Rest of Eastern Europe: Moldova.
8. Rest of European Free Trade Association: Iceland, Liechtenstein.
9. Rest of Europe: Andorra, Bosnia and Herzegovina, Faroe Islands, Gibraltar, Guernsey, Holy See (Vatican City State), Isle of Man, Jersey, Monaco, Montenegro, North Macedonia, San Marino, Serbia.
10. Rest of North America: Bermuda, Greenland, Saint Pierre and Miquelon.
11. Rest of North Africa: Algeria, Libya, Western Sahara.
12. Rest of Oceania: American Samoa, Cook Islands, Fiji, French Polynesia, Guam, Kiribati, Marshall Islands, Federated States of Micronesia, Nauru, New Caledonia, Niue, Northern Mariana Islands, Palau, Papua New Guinea, Pitcairn, Samoa, Solomon Islands, Tokelau, Tonga, Tuvalu, United States Minor Outlying Islands, Vanuatu, Wallis and Futuna Islands.
13. Rest of South Asia: Afghanistan, Bhutan, Maldives.
14. Rest of South African Customs Union: Eswatini, Lesotho.
15. Rest of Southeast Asia: Myanmar, Timor-Leste.
16. Rest of South America: Falkland Islands (Malvinas), French Guiana, Guyana, South Georgia and the South Sandwich Islands, Suriname.
17. Rest of Former Soviet Union: Turkmenistan, Uzbekistan.
18. Rest of the World: Antarctica, Bouvet Island, British Indian Ocean Territory, French Southern Territories.
19. Rest of Western Africa: Cape Verde, Gambia, Guinea-Bissau, Liberia, Mali, Mauritania, Niger, Saint Helena, Sierra Leone
20. Rest of Western Asia, Iraq, Lebanon, Palestinian Territory (Occupied), Syrian Arab Republic (Syria), Yemen.

#### D.3: List of sectors included in the model

Accommodation, Food & Service Activities; Air Transport; Beverages & Tobacco; Cane & Beet; Cattle; Cattle Meat; Chemical Products; Coal; Computer, Electronic & Optical Products; Construction; Dairy Products; Dwellings; Education; Electrical Equipment; Electricity; Fabricated Metal Products; Fibres crops; Fishing; Forestry; Furniture; Gas; Gas Manufacture; Human Health & Social Work; Information & Communication; Insurance; Iron & Steel; Land Transport; Leather Manufacture; Lumber; Machinery & Equipment; Manufacture of Textiles; Motor Vehicles; Non-Ferrous Metals; Oil; Oil Seeds; Other Animal Products; Other Business Services; Other Crops; Other Financial Intermediation; Other Food; Other Grains; Other Meat; Other Mineral Products; Other Mining Extraction; Other Services (Government); Other Transport Equipment; Paper & Paper Products; Petroleum & Coke; Pharmaceuticals; Processed Rice; Raw milk; Real Estate Activities; Recreation & Other Services; Rice; Rubber & Plastics Products; Sugar & Molasses; Veg & Fruit; Vegetable Oils; Warehousing; Water Supply; Water Transport; Wearing Apparel; Wheat; Wholesale & Retail Trade; Wool.

## Footnotes

1 See for example Felbermayr et al. (2020, 2021). D’Aguanno et al. (2021) show that re-shoring production may even increase economic volatility. The reader is referred to Miroudot (2020) for a more general discussion.

2 In March 2021, the ratification process of the Comprehensive Agreement on Investment (CAI) between the EU and China came to a halt following a series of sanctions and counter-sanctions amid the situation of the Uyghurs in Xinjiang province (European Parliament 2021). For an overview of the challenges currently inherent in trade and investment relations between the EU and China, the reader is referred to Garcia-Herrero et al. (2020).

3 NTBs include a wide spectrum of instruments such as import controls, state aid, public procurement policies and trade defence instruments (Ederington and Ruta 2016). They have been shown to have strong trade dampening effects (Kinzius et al. 2019; Ghodsi et al. 2017; Bratt 2017). In principle, decoupling can also be achieved by prohibitively high tariffs or firms’ autonomous decision to shift production back home.

4 The country group “US allies” is defined as the US, the EU27, Albania, Australia, Canada, Iceland, Japan, New Zealand, Norway, Philippines, South Korea, Taiwan, Turkey, and the United Kingdom.

5 A plethora of studies also investigates the impact of specific types of NTBs, such as sanitary and phytosanitary measures (Crivelli and Gröschl 2016), in particular against China (Beestermöller et al. 2017) or EU and US antidumping duties against China (Sandkamp 2020; Felbermayr and Sandkamp 2020; Sandkamp and Yalcin 2021). For an overview, the reader is referred to Ederington and Ruta (2016).

6 Our paper thus also relates to Chowdhry et al. (2022).

7 See Caliendo and Parro (2021) for a recent overview of this topic.

8 The factor is a composite of many factors, as for example labour, capital, and natural resources, which are all non-tradeable in our framework. In Sect. 5.3 we discuss the limitations to this approach.

9 The “phiness” of trade à la Baldwin et al. (2003).

10 Note that non-tariff barriers and iceberg trade costs are implemented in a similar fashion.

11 The productivity distribution is characterized by a location parameter $\lambda _{o}^{j}$ that varies by country and sector inducing *absolute* advantage, and a shape parameter $\theta ^{j}$ that varies by sector determining *comparative* advantage. $\theta ^{j}$ describes the elasticity of trade to trade costs.

12 In a first step, we compute a benchmark scenario by setting all aggregate trade imbalances $D_{d}$ to zero. This approach follows Ossa (2014) in order to account for the problem that the change in *real* income (Eq. 9) otherwise depends on what *nominal* units $D_{d}$ is measured in. The new equilibrium serves as a baseline for the counterfactual scenarios.

13 Non-tariff barriers are difficult to quantify due to a lack of sufficient global data coverage. We rely on the “exact hat algebra” approach, famously described by Dekle et al. (2008), and solve the model in changes. Therefore, we do not need to know the initial level of non-tariff barriers.

14 The paper thus differs from Felbermayr et al. (2020, 2021) who exclude oil and gas from the analysis.

15 Of course, in reality decoupling can be more subtle, leading to smaller reductions in imports. We discuss this in an extension in Sect. 5.

16 This is the most recent year for which input–output data for 141 countries/regions is available. We do not predict baseline values for some future year, since this would introduce additional margins of error.

17 Their GTAP 10 estimates are from October 2020 and can be found on their homepage.

18 Note that the latter number cannot be directly summed up by the values in Fig. 1 because the shares must be weighted by the relative GDP share in the coalition.

19 In the real world, one would observe a nominal depreciation of the Chinese Renminbi against the Euro following a fall in demand for Chinese currency. Within the model, a real depreciation takes place in the form of lower prices in China relative to the EU.

20 The model takes such general equilibrium effects into account through its balanced trade condition. Aggregate trade deficits/surpluses are assumed to remain constant. If China exports less to the EU following higher NTBs, this implies that China can either export more to the rest of the world (trade deflection) or reduce imports from both the EU and the rest of the world (trade depression). Both of these adjustment mechanisms can be observed in Scenario 1A.

21 Detailed results are provided by Table 3 in the Appendix.

22 These are mainly countries with which the US has some form of military alliance.

23 Within the model, barriers imposed on, say, EU exports to Russia have the same effect as barriers imposed by Russia on imports from the EU.

24 Detailed results are provided by Table 4 in the Appendix.

25 Detailed results are provided in Table 5 in the Appendix.

26 We increase NTBs in five specific GTAP sectors: Coal; Gas; Gas Manufacture; Oil; Petroleum & Coke. See Sect. D.3 for the full list of all sectors.
